# The Dopamine D1 Receptor Attenuates Titanium Particle-Induced Inhibition of Osteogenesis by Activating the Wnt Signaling Pathway

**DOI:** 10.1155/2023/6331650

**Published:** 2023-01-16

**Authors:** Chengcheng Feng, Yajun Li, Minhui Gu, Wenming Li, Yunshang Yang, Shuangshuang Chen, Yong Ma, Dechun Geng, Long Xiao, Zhirong Wang

**Affiliations:** ^1^Translational Medical Innovation Center, Zhangjiagang TCM Hospital Affiliated to Nanjing University of Chinese Medicine, Zhangjiagang 215600, China; ^2^Department of Orthopedics, Zhangjiagang TCM Hospital Affiliated to Nanjing University of Chinese Medicine, Zhangjiagang 215600, China; ^3^Laboratory of New Techniques of Restoration & Reconstruction of Orthopedics and Traumatology, Nanjing University of Chinese Medicine, Nanjing 210000, China; ^4^Department of Orthopedics, The First Affiliated Hospital of Soochow University, Suzhou 215006, China

## Abstract

Periprosthetic osteolysis (PPO), caused by wear particles, has become a major cause of joint replacement failure. Secondary surgery after joint replacement poses a serious threat to public health worldwide. Therefore, determining how to effectively inhibit wear particle-induced PPO has become an urgent issue. Recently, the interaction between osteogenic inhibition and wear particles at the biological interface of the implant has been found to be an important factor in the pathological process. Previous studies have found that the central nervous system plays an important role in the regulation of bone formation and bone remodeling. Dopamine (DA), an important catecholamine neurotransmitter, plays an integral role in the physiological and pathological processes of various tissues through its corresponding receptors. Our current study found that upregulation of dopamine first receptors could be achieved by activating the Wnt/*β*-catenin pathway, improving osteogenesis *in vivo* and *in vitro*, and significantly reducing the inhibition of titanium particle-induced osteogenesis. Overall, these findings suggest that dopamine first receptor (D1R) may be a plausible target to promote osteoblast function and resist wear particle-induced PPO.

## 1. Introduction

Artificial joint replacement is the most effective method to treat various end-stage bone and joint diseases and some bone tumor diseases [1]. However, it is predicted that by 2030, the demand for primary total hip arthroplasties is estimated to grow by 174% to 572,000. The demand for primary total knee arthroplasties is projected to grow by 673% to 3.48 million procedures [2]. According to a retrospective study of 23,269 hip revision patients [3], periprosthetic osteolysis (PPO) and subsequent aseptic loosening are the most common and potentially destructive long-term complications of total hip arthroplasty, accounting for more than 50% of all revision causes, which is the highest proportion. However, revision surgery is difficult due to trauma, high cost, and postoperative complications and will further increase the risk of further revision, resulting in a vicious cycle. Preventing and curing aseptic loosening of artificial joints is a great challenge in orthopedics [4–6].

The mechanism of long-term aseptic artificial joint loosening includes biological factors, micromotion of the prosthesis, stress shielding, and individual genetic differences [7]. Among them, PPO induced by wear particles is the main cause of aseptic loosening of prostheses, which directly affects the service life of prostheses [8]. Although macrophages and osteoclasts are considered to mediate bone destruction during PPO, previous studies have mainly focused on anti-inflammatory factors, osteoclasts, and improvements in biomaterials [9, 10]. However, inhibiting periprosthetic inflammation caused by wear particles and inhibiting osteoclast function are insufficient to treat osteolysis. Recently, many studies have shown that wear particle-induced inhibition of osteogenesis is an important cause of PPO [11]. Wear particles damage the expression of various collagens and alkaline phosphatase (ALP) activity in osteoblasts, inhibit osteogenic differentiation and mineralization, weaken the osteogenic potential of osteoprogenitor cells, and promote osteolysis [12–14]. In addition, wear particles can stimulate osteoblasts to secrete RANKL, promote osteoclast activation, and further exacerbate osteolysis [15]. Therefore, reversing osteoclast inhibition and promoting the differentiation and proliferation of osteoblasts may be a promising treatment strategy for PPO.

The central nervous system plays a role in the regulation of bone formation and bone remodeling [16]. The central nervous system influences bone physiology through the peripheral nervous system, pituitary-derived hormones, and other brain-derived molecules [17]. Dopamine (DA) is an important catecholamine neurotransmitter that is widely present in peripheral and central tissues. DA plays an important role in the physiological and pathological processes of various tissues through its corresponding receptors [18]. A lack of DA can lead to neurological and mental disorders, such as Parkinson's disease and depression [19]. Studies have shown that individuals with these two diseases have a higher risk of osteoporotic fractures than the general population, suggesting that the concentration of DA may affect bone mass [20]. Buckley et al. [21] reported an inhibitory effect of dopamine receptor antagonist on bone formation via the suppression of osteoblastic cell functions *in vivo* and *in vitro*.

It has been reported that DA can inhibit bone resorption through the NFATc-1 and c-fos signaling pathways and interact with receptors on osteoclasts [22, 23]. Our previous studies also showed that DA treatment can prevent osteolysis caused by wear debris by inhibiting RANKL-dependent osteoclast formation and the inflammatory response mediated by DA-like receptors [24]. Recent studies have shown that DA can affect the proliferation and osteogenic differentiation of BMSCs through dopamine first receptor (D1R) [25, 26]. Whether the regulation of DA receptors can alter bone formation in titanium-induced osteolysis is unclear.

**Scheme 1:**
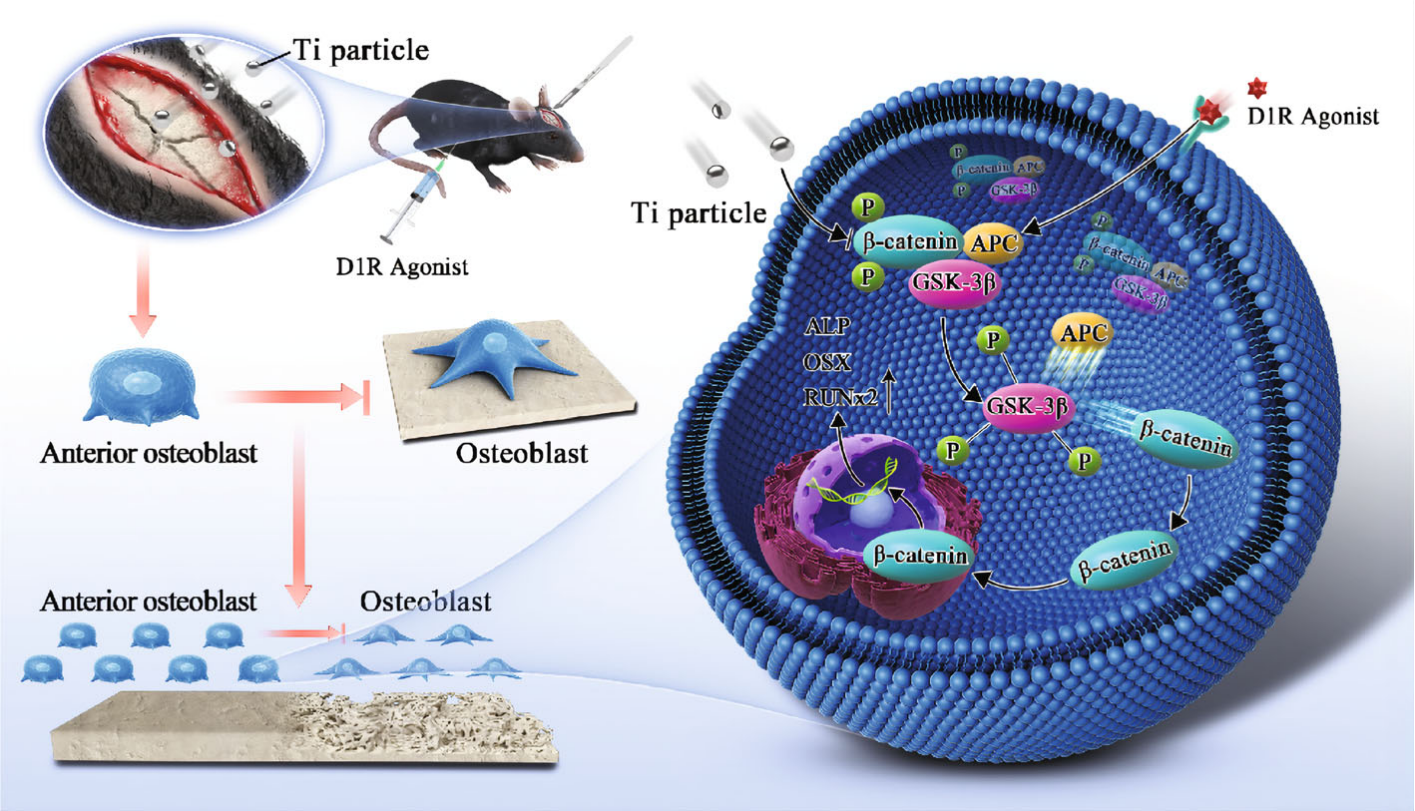
D1R activation alleviates the osteogenic inhibition caused by wear granules on the mouse skull and alleviates the inhibition of osteoblast differentiation induced by titanium granules. D1R activation can promote the expression of the osteoblast-related proteins ALP, OSX, and Runx2 by activating the Wnt pathway and abrogate the inhibition of osteoblast differentiation induced by titanium particles.

We investigated the role of DA receptors in titanium particle-induced inhibition of osteoblast function in a mouse skull osteolysis model and wear particle-induced osteoblast differentiation model. Our results indicated that the expression of D1R was inhibited in titanium particle-induced osteolysis, while D1R activation could reduce bone loss caused by wear particles. In addition, we observed that D1R activation could promote osteoblast differentiation through the Wnt signaling pathway (Scheme 1). In conclusion, D1R is a suitable target for the prevention and treatment of PPO.

## 2. Materials and Methods

### 2.1. Preparation of Titanium Particles

Titanium particle (00681, Walhill, Massa, USA) parameters: average particle size is 3.32 ± 2.39 mm with more than 90% particle size <3.6 mm and 50% particle size <1.6 mm. Endotoxin on the surface of titanium particles was removed by 12 h high temperature calcination and 75% ethanol washing for 48 h. The removed concentration of endotoxin was determined by a Limulus Amoebocyte Lysate assay detection kit (LAL, Biotaker, Whorksville, MD, USA), and 50 *μ*l and 0.1, 0.25, 0.5, 0.5, and 1.0 EU/ml of endotoxin standard and titanium particle suspension with 0.1 mg/ml were placed in a sterile reaction tube. The LAL and matrix solutions preheated to 37°C were successively added to the reaction tube, and the reaction was terminated after 10 min. Reaction system to the samples absorbance was measured in 200 *μ*l of the supernatant at 405 nm in 96-well plates. Only titanium particle samples with measured endotoxin levels below 0.02 EU/ml were selected for this experiment.

### 2.2. Cell Culture and Osteoblast Differentiation

The MC3T3-E1 cells (Rockville, Maryland, USA) and BMSC cells (MUBMX-01001, Cyagen, Guangzhou, China) were grown in medium containing 10% FBS (16140071, Rockville, USA). Osteogenic induction medium (DMEM, SH30022.01, Cytiva, Pittsburgh, USA) supplemented with 10% FBS, 100 nm dexamethasone (D1756, Sigma-Aldrich, MO, USA), 10 mm *β*-glycerophosphate (G9422, Sigma-Aldrich, MO, USA), and 50 mm vitamin C (PHR1008, Sigma-Aldrich, MO, USA). To mimic the osteolytic microenvironment, 5 *μ*g/cm^2^ of titanium particles were added to the culture medium. Then, the cells were pretreated with either a D1R agonist (10 *μ*M, SKF38393) or a D1R agonist (10 *μ*M) plus an inhibitor (0.2 nm, SCH23390).

### 2.3. Cell Viability Assay

MC3T3-E1 cells were incubated for 12 h in 96-well plates in order to make sure cell adhesion. To evaluate the cytotoxicity, different concentrations of D1R agonists and D1R inhibitors and ICG-001 (847591-62-2, Sigma-Aldrich, Missouri, USA) were added and incubated for 1, 3, and 5 days. Then, we used the Cell Counting Kit-8 (CCK-8; C0038, Beyotime, Shanghai, China). The absorbance was measured at 450 nm using an enzyme marker (BioTek, Winooski, VT, USA) after 1 h incubation at 37°C and 5% CO_2_. The inhibition curve of MC3T3-E1 was drawn with GraphPad Prism 7.

### 2.4. ALP Staining

Briefly, BMSCs were washed with PBS gently after 10 days of osteogenic induction. Subsequently fixed in 4% paraformaldehyde for 30 min and washed with PBS, then BCIP/NBT working solution (C3206, Beyotime, Shanghai, China) was added to the well plate and incubated at 37 degrees for 30 min, and this was observed under a microscope.

### 2.5. Alizarin Red S (ARS) Staining

BMSCs were cultured in osteogenic medium for 21 days. Then, fixed with 4% paraformaldehyde for 30 min and washed three times with ddH_2_O, the alizarin red S solution (G1450, Solarbio, Beijing, China) was added and incubated at 37 degrees for 30 min. The staining was observed under a microscope.

### 2.6. Western Blot Analysis

MC3T3-E1 cells were induced in osteogenic induction medium for 3 days. Cells were lysed by radioimmunoassay (RIPA; Beyotime), centrifuged and the supernatant was collected, and the total protein concentration was quantified by BCA kit (Beyotime). Total protein (20 *μ*g) was separated by SDS-PAGE (New Cell and Molecular Biotechnology Limited, Suzhou, China) and transferred to a nitrocellulose membrane. The cells were incubated with the appropriate primary antibody for 16 h at 4°C after blocking, and the antibody of Runx2 (1 : 1000), Osterix (1 : 1000), total GSK-3*β* (1 : 1000), pser 9-gsk-3 *β* (1 : 1000), and *β*-catenin (1 : 5000) was purchased from Abcam (ab264077, ab209484, ab93926, ab107166, and ab32572). Membranes were washed with TBST (BP-G0004, CWBiotech, Beijing, China), then incubated with secondary antibody and observed with chemiluminescent HRP substrate (WBKLS0500, Millipore Corp.).

### 2.7. Immunofluorescence Staining

BMSC cells were seeded in 24-well plates and then cultured in osteogenic differentiation medium with or without titanium particles. We fixed and permeabilized the cells. Then, we used anti-*β*-catenin (1 : 200) to label the target protein. Subsequently, fluorescent red was marked with the molecular probe Alexa Fluor® 555 fluorescein (1 : 1000, # 4417 Cell Signaling Technology, Danfoss, USA). Finally, the well plate was covered with a plate solution containing DAPI (Beyotime). The staining was observed under a microscope.

### 2.8. Animal Research and Drug Application

As mentioned previously, a titanium-induced mouse skull model was established. Briefly, 8 weeks male C57BL/6 mice (Shanghai J&J Laboratory Animal Co., Ltd.) were divided randomly into 4 groups (*n* = 10/group): (1) sham operation group, saline treatment; (2) model group, saline treatment and 20 mg of titanium particles; (3) D1R receptor agonist treatment group, model mice were given 10 mg/kg agonist every day; (4) D1R agonist+inhibitor treatment group, model mice were given 10 mg/kg D1R agonist daily plus 10 mg/kg D1R inhibitor. After anesthesia and skin preparation, a longitudinal incision was made at the top of the mouse head, and the skull was exposed. The periosteum and skull were destroyed along the sagittal suture. The sham group had the incision closed without additional intervention. The other two groups received 20 mg of titanium particles subperiosteum. The D1R agonists and inhibitors were from Sigma-Aldrich (St. Louis). The D1R receptor agonist treatment group received the agonist in a DMSO solution, and the D1R inhibitor treatment group received the same amount of agonist plus the inhibitor in a DMSO solution. Two days later, the model group, D1R agonist group, and D1R agonist+inhibitor group were intraperitoneally injected with normal saline, the D1R agonist or the D1R agonist plus the inhibitor once per day for one week. Skulls, livers, and kidneys were gathered for further analysis.

### 2.9. Micro-CT Analysis

One week later, the mice were killed, and the skulls of 5 mice/group were fixed with 4% paraformaldehyde, and micro-CT (SkyScan 1176, SkyScan, Kontich, Belgium) method was used to measure the erosion of the skull surface. The 3D images were reconstructed and analyzed quantitatively by SkyScan software which located a region of interest of equal area (3 mm in diameter) in the center of each skull. The results were analyzed using SkyScan software.

### 2.10. Histological Analysis

After micro-CT analysis, the skull decalcification was conducted with 10% EDTA and embed sections with paraffin. After sectioning, it is used for histological analysis. We chose to stain the sections with hematoxylin and eosin (H&E) and Masson's trichrome staining, according to the correct instructions

### 2.11. Immunofluorescence Staining

In brief, tissue sections were dewaxed, extracted for antigen, and blocked for 60 min with 2% BSA. Then, primary antibodies were inserted and incubated at 4°C overnight, including Runx2 (ab192256) and Osterix (ab209484, all from Abcam). Sections were then closed in the dark for 60 min with the corresponding fluorescent secondary antibodies (Alexa Fluor® 555 and 488 (Abcam)). In the end, the staining was observed with a fluorescence microscope (Zeiss).

### 2.12. Statistical Analysis

The data were expressed as mean ± standard deviation (SD). GraphPad Prism 7 was used for statistical analyses. The D'Agostino-Pearson analysis was used after verifying the normal distribution. One-way ANOVA and Tukey's multiple comparison test were used to analyze statistical significance. The value of *P* < 0.05 was deemed statistically significant.

## 3. Results

### 3.1. Titanium Particles Inhibit the Differentiation of Osteoblasts and D1R Expression

**Figure 1:**
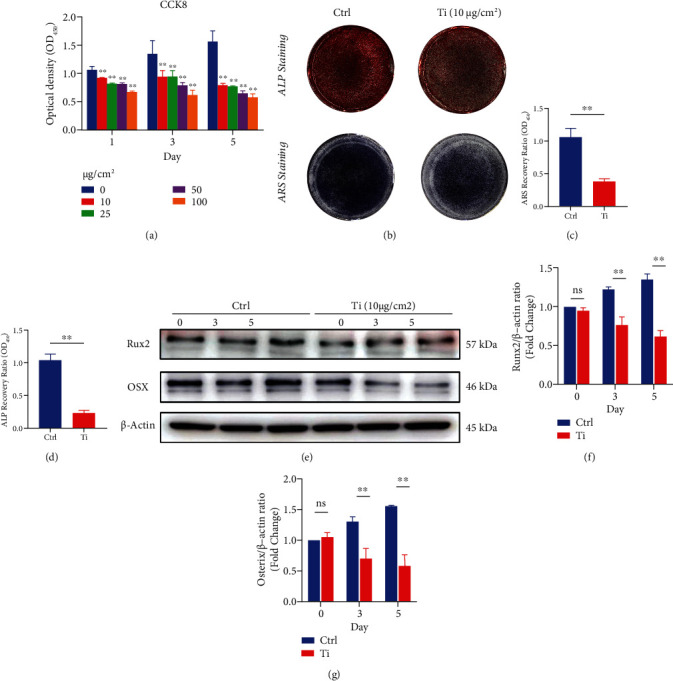
Titanium particles inhibit the differentiation of osteoblasts *in vitro*. (a) MC3T3-E1 cell viability was measured by the CCK-8 assay. (b) Representative images of ALP and ARS staining *in vitro*. (c, d) Quantification of ALP activity and bone mineralization in MC3T3-E1 cells in each group. Note: *n* = 3 per group; ^∗^*P* < 0.05 and ^∗∗^*P* < 0.01 compared with the Ctrl group (without titanium particle intervention). (e) Representative images of western blots probed with antibodies against OSX and Runx2. (f, g) Quantification of OSX and Runx2 protein levels. *n* = 3 per group. NS: not statistically significant; ^∗^*P* < 0.05 and ^∗∗^*P* < 0.01 vs. the ctrl group at the same time point.

To investigate the effect of DA receptors on wear particle-induced osteoblast inhibition, we treated MC3T3-E1 cells with osteogenesis induction medium containing 10, 25, 50, and 100 *μ*g/cm^2^ titanium granules, and the CCK-8 results revealed that titanium particles inhibited the osteogenic differentiation of MC3T3-E1 cells dose dependently (Figure 1(a)). To exclude inhibition of MC3T3-E1 cells due to limited growth space caused by excessive levels of titanium particles, we selected 10 *μ*g/cm^2^ titanium particles as the experimental concentration. To further investigate the effects of D1R on titanium particle-induced osteoblast inhibition, MC3T3-E1 cells were treated with osteogenic induction medium supplemented with 10 *μ*g/cm^2^ titanium particles. The ALP and ARS staining results showed that titanium particle treatment significantly inhibited the ALP activity and bone mineralization in MC3T3-E1 cells (Figures 1(b)–1(d)). Moreover, western blot analysis showed that 10 *μ*g/cm^2^ titanium particle treatment markedly decreased the expression of the osteogenic proteins Osterix and Runx2 compared to those in the control group (Figure 1(e)). Quantitative analysis of the relative gray levels provided validation of these changes (Figures 1(f) and 1(g)).

**Figure 2:**
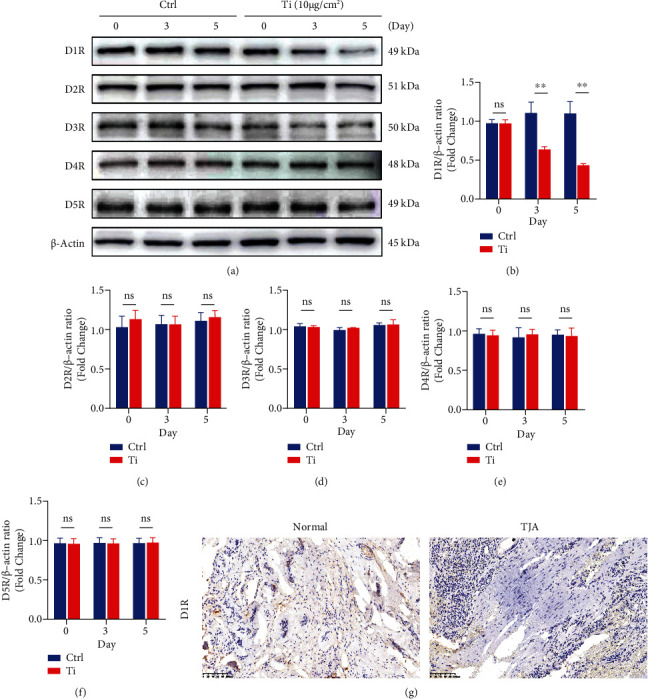
Titanium particles inhibit D1R expression *in vitro* and *ex vivo* of human. (a) Representative images of western blots probed with antibodies against D1R, D2R, D3R, D4R, and D5R. (b–f) Quantification of D1R, D2R, D3R, D4R, and D5R protein levels. *n* = 3 per group. NS: not statistically significant, ^∗^*P* < 0.05 and ^∗∗^*P* < 0.01 vs. the ctrl group at the same time point. (g) Representative images showing IHC staining of D1R in human synovial specimens. Scale bar: 50 *μ*M.

We further investigated the effect of D1R on titanium particle-induced osteoblast inhibition, and by western blotting, we found that D1R expression was major reduced in the presence of titanium particles, while d2-5R expression was independent of the presence of titanium particles (Figure 2(a)). Quantitative analysis of the relative gray levels further verified these changes (Figures 2(b)–2(f)). These results suggest that D1R may be associated with wear-induced osteoblast inhibition. Interestingly, in the clinical specimens, by immunohistochemical staining, we found that the expression of D1R in the synovial specimens of the arthroplasty patients was significantly lower than that in the normal synovial specimens (Figure 2(g)).

### 3.2. D1R Agonist Treatment Attenuates Titanium-Induced Osteogenesis *In Vitro*

**Figure 3:**
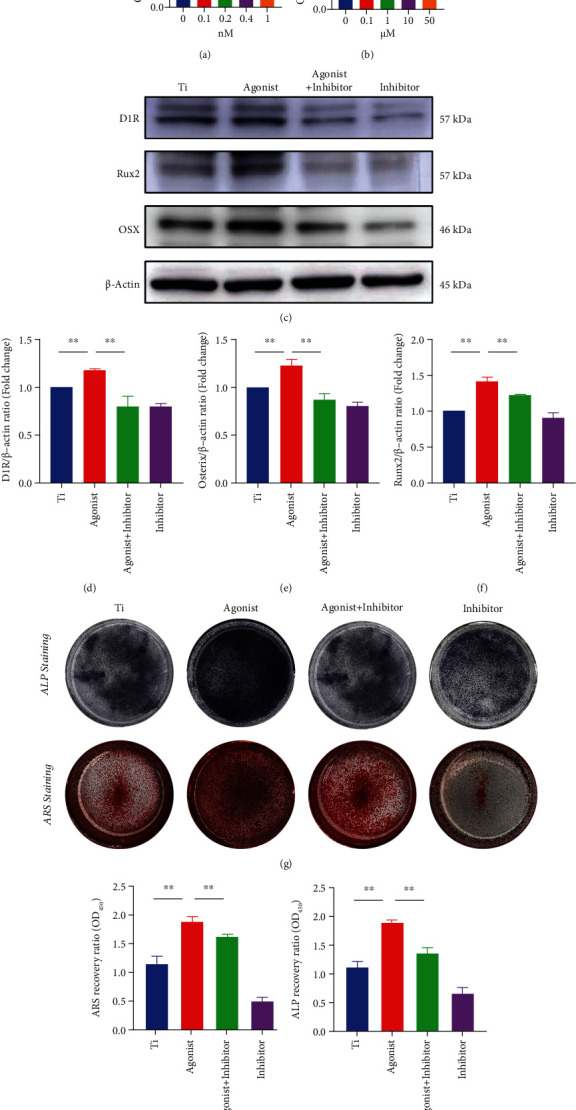
D1R activation abrogates osteogenesis inhibition caused by titanium particles *in vitro*. (a, b) Cell viability of MC3T3-E1 cells treated with D1R agonists and inhibitors was measured by CCK-8 assays. (c) Representative western blots probed with antibodies against OSX, Runx2, and D1R. (d–f) Quantification of OSX, Runx2, and D1R protein levels. *n* = 3 per group. NS: no statistical significance, ^∗^*P* < 0.05 and ^∗∗^*P* < 0.01 compared to the Ti group (titanium particle intervention group). (g) Representative images of ALP and ARS staining *in vitro*. (h, i) Quantitative analysis of ALP activity and bone mineralization in BMSC cells in each group. Note: *n* = 3 per group; ^∗^*P* < 0.05 and ^∗∗^*P* < 0.01 compared with the Ti group.

To further examine the role of D1R in wear particle-induced inhibition of osteoblastogenesis and bone construction, we used the D1R-specific agonist SKF38393 and inhibitor SCH23390 to upregulate and downregulate the expression of D1R, respectively. A CCK-8 assay was performed. The results showed that 10 *μ*M SKF38393 and 0.2 nM SCH23390 did not affect cells (Figures 3(a) and 3(b)). As Figure 3(c) shows, SKF38393 protein promoted the expression of D1R protein, while SCH23390 had a repressive effect on D1R protein expression. Then, 10 *μ*M SKF38393 and 0.2 nM SCH23390 were added to the osteoblast medium containing titanium particles one day later. Western blot analysis showed that titanium particles in osteoblasts significantly downregulated the osteogenic transcription factors OSX and Runx2. In contrast, the changes in protein levels were reversed by SKF38393, but the effect was inhibited by the addition of SCH23390 (Figures 3(d)–3(h)). The ALP staining showed that titanium particles could depress the osteogenic differentiation of MC3T3-E1 cells, while SKF38393 (a D1R agonist) mitigated the inhibition of osteogenesis caused by titanium particles, and this relief was weakened by the addition of SCH23390 (a D1R inhibitor). Consistent with this finding, ARS staining showed that SKF38393 (D1R agonist) treatment stimulated cell mineralization by approximately 172.7% compared with that in cells treated with titanium particles only (Figures 3(g)–3(i)). In summary, these data indicate that D1R agonists attenuate the titanium particle-induced inhibition of osteoblast differentiation *in vitro*, whereas D1R inhibitors attenuate the agonist effect.

### 3.3. D1R Activation Attenuates Titanium Particle-Induced Osteogenic Inhibition by Activating the Wnt Signaling Pathway *In Vitro*

**Figure 4:**
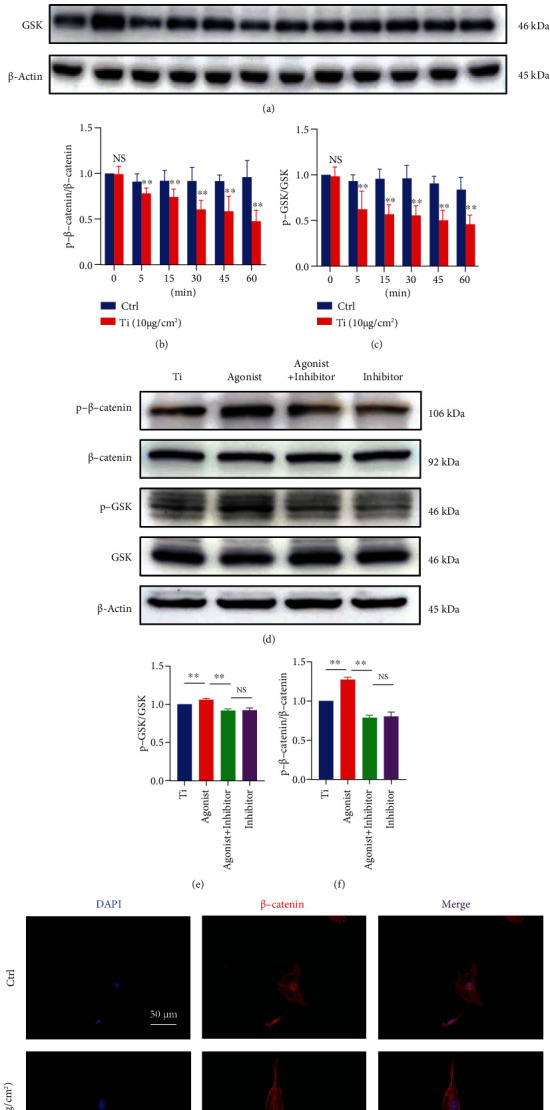
Activation of D1R alleviated the inhibition of osteoblast-related pathway protein expression caused by titanium particles. (a, d) Western blots were probed with antibodies against the osteoblast-associated proteins GSK, p-GSK, *β*-catenin, and p-*β*-catenin. (b, c) Temporal analysis of osteoblast-related protein expression. (e, f) Quantitative analysis of the expression of the osteoblast-related proteins GSK, p-GSK, *β*-catenin, and p-*β*-catenin in the presence of D1R agonists and/or inhibitors. Note: ^∗^*P* < 0.05 and ^∗∗^*P* < 0.01 vs. titanium particle inducer group (without treatment with D1R agonists and/or inhibitors). (g) D1R immunofluorescence staining. Scale bar: 50 *μ*M.

It is well known that the Wnt pathway plays an indispensable role in the differentiation of osteoclasts [27]. In this study, 10 *μ*g/cm^2^ titanium particles were administered to MC3T3-E1 cells, the expression of *β*-catenin was measured at 0, 5, 15, 30, 45, and 60 min, and we found that titanium particles inhibited the expression of *β*-catenin in cells beginning at 5 min; there was a statistically significant difference compared with that in the control group, and the most significant inhibition occurred at 60 min (Figures 4(a)–4(c); *P* < 0.01). Furthermore, we used immunofluorescence staining to verify these changes. As shown in Figure 4(g), in the 0 g/cm^2^ group, the fluorescence intensity of *β*-catenin in the nucleus was significant, whereas titanium particles (10 g/cm^2^) significantly reduced the fluorescence intensity of *β*-catenin in the nucleus.

We then investigated the protein expression of *β*-catenin in the presence of D1R agonists and inhibitors and 10 g/cm^2^ titanium particle culture conditions by immunoblot analysis. In contrast to that of cells treated with titanium particles alone, the Wnt level in cells treated with the DIR agonist was significantly increased, and the expression of *β*-catenin was increased (Figures 4(d)–4(f); *P* < 0.01). However, the addition of the inhibitor significantly reduced the expression level of *β*-catenin.

**Figure 5:**
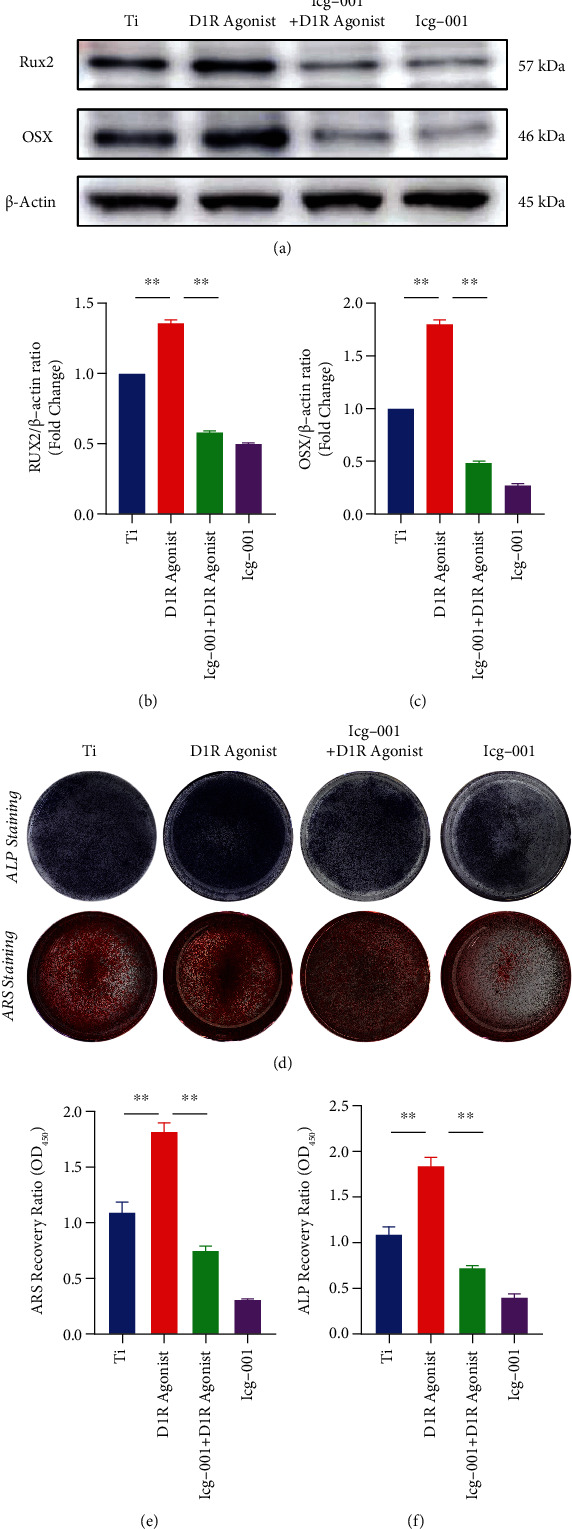
D1R upregulation attenuates titanium particle-induced osteogenic inhibition by activating the Wnt signaling pathway *in vitro*. (a) Representative images of western blots probed with antibodies against OSX and Runx2 in the presence of a Wnt pathway inhibitor (icg-001). (b-c) Quantification of OSX and Runx2 protein levels. *n* = 3 per group. NS: Not statistically significant, ^∗^*P* < 0.05, ^∗∗^*P* < 0.01, vs. titanium particle inducer group (without treatment with D1R agonists and/or icg-001). (d) Representative images of ALP and ARS staining *in vitro*. (e-f) Quantitative analysis of ALP activity and bone mineralization in BMSC cells in each group. Note: *n* = 3 per group; ^∗^*P* < 0.05, ^∗∗^*P* < 0.01 compared with the titanium particle inducer group (without treatment with D1R agonists and/or icg-001).

Icg-001 (Selleck) was used as a specific inhibitor of the Wnt/*β*-catenin protein signaling pathway to validate the role of the Wnt/*β*-catenin protein signaling pathway. The cytotoxic effect of ICG-001 was determined by CCK-8 cell viability assay. The results showed that the effect of ICG-001 at a concentration of 20 *μ*M (Figure 5(a)) on cell viability. Western blot analysis showed that ICG-001(20 *μ*M) significantly inhibited *β*-catenin expression (Figures 5(b) and 5(c)), and compared to titanium particle treatment, the D1R agonist significantly promoted the expression of osteogenic markers; however, the expression of osteogenic markers was decreased by icg-001 treatment (Figures 5(d)–5(f)). ALP staining showed that the D1R agonist could alleviate the inhibitory effect of titanium particles on ALP activity, and ICG-001 attenuated the effects of the D1R agonist. In addition, ARS staining confirmed that the D1R agonist alleviated osteogenic mineralization caused by the titanium particles, and that, ICG-001 further counteracted the effects of the D1R agonist (Figures 5(g)–5(i)). These findings suggest that D1R activation attenuates the inhibition of osteogenesis induced by titanium particles via the Wnt/*β*-catenin signaling pathway.

### 3.4. D1R Activation Promotes Bone Formation and Attenuates Titanium-Induced Bone Destruction in a Mouse Model

**Figure 6:**
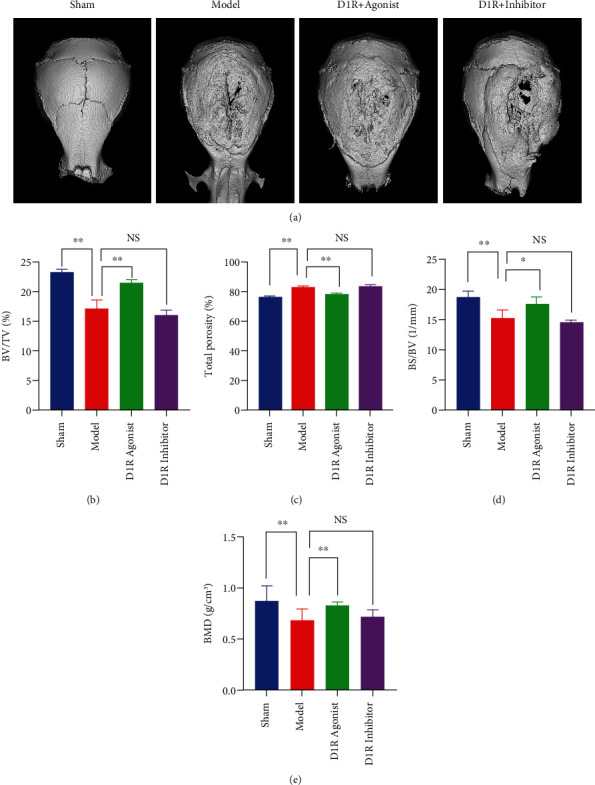
D1R activation alleviates titanium particle-induced osteolysis *in vivo*. (a) Representative 3D *μ*CT images of the mouse skull in the sham group, model group, D1R agonist group, and D1R inhibitor group. (b–e) Bone mineral density (BMD, in g/mm^3^) and comparative analysis of bone structural parameters, such as the ratio of bone volume to total volume (BV/TV, in %), ratio of bone surface to bone volume (BS/BV, in 1/mm), and ratio of bone surface to total volume (BS/TV, in 1/mm). Note: *n* = 5 per group; ^∗∗^*P* < 0.01 vs. the model group.

A titanium particle-induced murine calvaria osteolysis model was established, and the results showed that two weeks after the implantation of titanium particles, bone formation was decreased and bone destruction was increased significantly. Titanium particles enhanced bone loss in the mouse skull, and D1R agonist treatment significantly reduced this loss (Figure 6(a)). Compared with that in the sham operation group, the BMD in the model group was significantly decreased (102.6 ± 4.526 mg/mm^3^). Compared with that in the model group, the decrease in BMD in the D1R agonist group (119.8 ± 2.351 mg/mm^3^) was significantly abrogated; compared with that in the agonist treatment group, the BMD in the D1R agonist+inhibitor group was significantly decreased (100.2 ± 3.57 mg/mm^3^) (Figure 6(b)). In addition, other parameters, such as BV, BV/TV, and pore number, indicated that the D1R agonist could alleviate titanium-induced osteolysis, while the D1R inhibitor could block the protective effect of the agonist against osteolysis (Figures 6(c)–6(e)).

**Figure 7:**
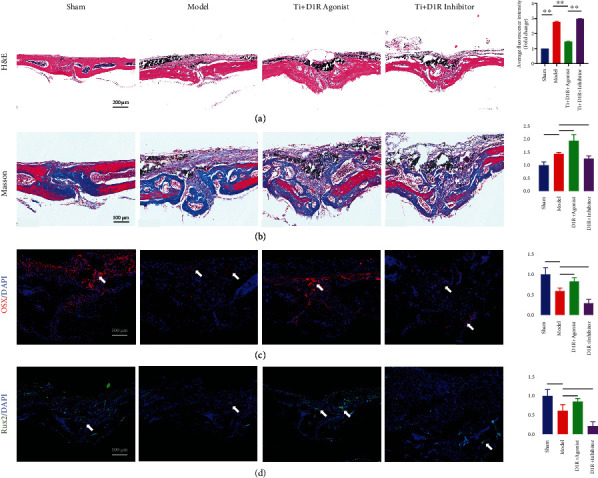
Activating D1R relieves mouse osteolysis caused by titanium particles and relieves osteogenesis inhibition. (a, b) Representative paraffinized sections were stained with H&E and Masson's trichrome staining and their quantification. (c, d) Representative images and quantization showing immunofluorescence staining of the osteoblast-related proteins OSX and Runx2 in skull sections of mice in the sham group, model group, D1R agonist group, and D1R inhibitor group.

In order to further observe the degree of bone erosion and osteogenic ability in different groups, both histological H&E and Masson's staining were performed. H&E staining showed widespread erosion and fibrotic hyperplasia on the skull surface in the model group. D1R agonist treatment significantly reduced bone erosion and fibrosis. However, treatment with D1R inhibitors did not provide remarkable protection against titanium particle-induced bone erosion and fibrosis (Figure 7(a)). Next, Masson's staining indicated that the development of new immature collagen fibers in the skull of the model group of mice was significantly reduced, but D1R agonist treatment enhanced vigorously the formation of collagen fibers. However, in the D1R inhibitor-treated group, there was no apparent increase in collagen fibril formation (Figure 7(b)). Those outcomes suggested that D1R activation protected mouse cranial bone from titanium particle-induced attack and facilitated osteogenic capacity *in vivo*.

To further verify whether D1R agonist treatment alleviated titanium-induced osteolysis, we examined the expression of osteogenic-associated transcription factors using IHC staining. The total bone composition capacity of the D1R agonist treatment was significantly higher than that of the titanium particle alone intervention group, as evidenced by the increased expression of the osteogenesis-related transcription factors Runx2 and OSX (Figures 7(c) and 7(d)). The number of IHC positive cells was significantly lower in the model group compared to the sham and D1R agonist-treated groups. These results indicate that titanium granules can inhibit the expression of osteogenesis-related transcription factors, while D1R agonist treatment alleviates the inhibitory effect of titanium granules on osteoblasts.

## 4. Discussion

Osteoblasts are the major functional cells associated with bone formation and are involved in the synthesis, secretion, and mineralization of the bone matrix. Normal bone metabolism depends on the dynamic balance between bone formation and bone degradation [28]. Numerous studies have clearly shown that various types of wear particles have significant effects on the function of osteoblasts [29]. Proinflammatory and osteoclastic factors secreted by macrophages, fibroblasts, lymphocytes, and osteoclasts may upset the equilibrium between bone formation and bone resorption, leading to PPO [30]. The previous studies have concentrated on anti-inflammatory factors, osteoclasts, and improvements in biomaterials [9, 29]; however, inhibiting inflammation around the prosthesis caused by wear particles has been shown to be insufficient for treating osteolysis. As the main components of bones, the osteoblast type I precollagen gene, calcium deposition, and ALP activity are decreased by wear particles, which is another important cause of PPO [31]. Nam et al. [29] found that in osteoblasts exposed to wear particles, inflammation, and bone resorption factor expression levels were increased, and bone formation-related factor expression levels were decreased, resulting in decreased osteogenesis. In this study, we found that in the osteolytic model, the bone density of the skull was reduced, the bone trabeculae were sparse, and the bone mass was reduced. ALP analysis and ARS staining confirmed a reduction in bone formation during bone dissolution induced by wear particles, which is consistent with our previous report [26]; osteogenesis inhibition is one of the main causes of PPO, and reducing hypogenesis around prostheses induced by wear particles is a key factor in the prevention and treatment of PPO.

The central nervous system and neurotransmitters have a major role in the regulation of the skeletal system [17]. The receptors for a wide variety of neurotransmitters are voiced on the surface of osteoblasts, which indicates the importance of this neurobone interaction [32]. In the past 15 years, studies combining mouse and human genes have shown that the brain is a powerful regulator of bone growth. DA is an important neurotransmitter in the central nervous system. It has been confirmed that the differentiation of osteoblasts involves neurotransmitters and plays a role in bone development and repair [33]. Studies have shown that individuals with diseases caused by DA deficiency have higher risk of developing osteoporotic fractures than those in the general population, suggesting that the concentration of DA may affect bone mass. Wang et al. [25] showed that D1R activation mitigated the reduction in DEX-induced osteogenic differentiation in BMSCs. This finding is consistent with our results; we found that D1R was widely expressed in bone tissue and could affect the proliferation and differentiation of osteoblasts. Interestingly, in our previous study, we showed that DEX reduced the expression of D1R, thereby inhibiting osteogenic differentiation, while D1R activation weakened DEX-mediated inhibition of bone formation [26]. In this study, we found that after D1R activation, the number of osteoblasts and the degree of differentiation improved. D1R activation may promote the function of osteoblasts, alleviate the inhibition of osteogenesis, and improve the destruction of bone by titanium particles.

The Wnt/*β*-catenin signaling pathway is a critical pathway in bone formation and plays a crucial role in the promotion, osteogenic differentiation, and calcification of human bone marrow mesenchymal stem cells [34, 35]. In our study, we found that this pathway was inhibited by titanium particles, while D1R activation could promote nuclear translocation and reduce the inhibitory effect of titanium particles on osteoblasts. Singh et al. [36] showed that D1R activates antidepressant-like effects via activation of the Wnt/*β*-catenin pathway in a rat model of Parkinson's disease. Previous studies reported that acetyl-11-keto-*β*-boswellic acid (AKBA) could promote the proliferation, differentiation, and maturation of osteoblasts through the activation of the Wnt/*β*-catenin signaling pathway [37]. We also observed that inhibiting Wnt/*β*-catenin decreased the effect of D1R on osteoblasts. These results further confirm that D1R may play a regulatory role via the Wnt/*β*-catenin pathway.

In this study, we used a mouse osteolysis model. Compared with implanting long bones with clinical prostheses, the mouse skull is a lamellar bone, which is different from that in clinical practice. However, it is the fastest and most common osteolysis model and can accurately simulate the biological process of clinical prosthesis loosening. In the following studies, we will continue to investigate these questions in depth and further elucidate the role of D1R in the regulation of titanium particle-mediated bone remodeling using large animal or primate femurs as models. In the present model, titanium particles were administered once; next, we will use a sustained-release device to slowly release wear particles to simulate clinical biological processes and verify our results. We used peptide particles, while clinical wear particles are mostly polyethylene particles. Moreover, the density of polyacetylene is low, and it easily floats on the surface of culture medium or PBS, which was not conducive for use as a model in the current study. Titanium particles not only have higher operability *in vitro* but also have been confirmed to produce similar biological effects as polyethylene particles. Furthermore, our model also confirmed that titanium particles could successfully lead to osteolysis. We will subsequently verify our conclusions with polyethylene particles. Osteoclast activation and local inflammatory response play a nonnegligible role in titanium particle-induced osteolysis. Titanium particles can cause monocytes/macrophages to trigger the release of proinflammatory factors, initiate an inflammatory cascade response, release osteoclasts, promote osteoclast recruitment, migration, and differentiation, and ultimately activate bone resorption, leading to osteolysis [38]. Panicker et al. [39] reported that DA inhibited NLRP3 inflammasome activation via D1R, thereby regulating inflammation. However, the present study cannot directly elucidate the relationship between inflammatory D1R in titanium particle-induced osteolysis, so we will carry out further studies and experiments.

## 5. Conclusion

In conclusion, this study shows that D1R activation can alleviate osteogenic inhibition caused by titanium particles through the Wnt/*β*-catenin signaling pathway and promote the differentiation of osteoblasts. To understand the direct regulatory role of D1R on osteoblasts and its mechanism will contribute to providing a new therapeutic strategy for wear particle-induced osteolysis.

## Data Availability

The data that support the findings of this study are available from the corresponding author upon reasonable request.

## Abbreviations

D1R: Dopamine 1 receptor
ALP: Alkaline phosphatase
ARS: Alizarin red S
Runx2: Runt-related transcription factor 2
GSK-3*β*: Glycogen synthase kinase-3*β*
ICG-001: Indocyanine green-001
IHC: Immunohistochemical
BV/TV: Bone volume per tissue volume
BS/BV: Bone surface/bone volume
BS/TV: Bone surface/total volume
Tb.N: Trabecular number
Tb.Th: Trabecular thickness
PPO: Periprosthetic osteolysis
RIPA: Radio immunoprecipitation assay.
